# Supplementary material for: Percutaneous Closure of Mitral Paravalvular Leak: Long-Term Results in a Single-Center Experience
Source: J Clin Med. 2022 Aug 18;11(16):4835. doi: 10.3390/jcm11164835 (PMC9409651; doi:10.3390/jcm11164835)

# PERCUTANEOUS CLOSURE OF MITRAL PARAVALVULAR LEAK: LONG-TERM RESULTS IN A SINGLE-CENTER EX-PERIENCE

## SUPPLEMENTARY MATERIAL

**Supplementary Table S1: Predictors of procedural success:**

| Factor | P value | |
|---|---|---|
| | Univariant analysis | Multivarian analysis |
| NYHA I-II vs III-IV | 0.046 | 0.093 (NS) |
| HF vs HA indication | 0.002 | 0.054 |
| Transfusion (yes/no) | 0.000 | 0.085 (NS) |
| Location (multiple vs no multiple PVL) | 0.009 | 0.030 |
| More than 2 previous surgeries | 0.015 | NS |
| Basal hemoglobin | 0.063 | NS |
| Basal LDH | 0.000 | NS |
| Pulmonary hypertension | 0.135 | - |

Univariate analysis: Pulmonary hypertension, more than 2 previous surgeries, basal NYHA, HF vs HA indication, blood transfusion (yes/no), location (multiple vs no multiple PVL), basal hemoglobin, basal LDH.

**Supplementary Table S2: Predictors of major adverse cardiovascular events (MACE) during long-term follow-up:**

| | P value | HR | 95% Conf. Interval |
|---|---|---|---|
| CKD | 0.043 | 2.2 | 1.0 – 4.6 |
| 3 months HF previous admission | 0.000 | 5.7 | 2.3 – 13.8 |
| First proc. Success | 0.002 | 0.1 | 0.0 – 0.5 |
| Improve NYHA class at follow-up | 0.011 | 0.4 | 0.2 – 0.8 |
| HF vs HA indication | 0.000 | 5.9 | 2.9 – 12.2 |

Univariate analysis: Sex, previous CAD, CKD, basal NYHA, 3 months HF previous admission, previous red blood cells transfusion (yes/no), basal hemoglobin, basal LDH, STS score, EuroScore I, HF vs HA indication, PVL location, technical success, procedural

success, procedural complications, in-hospital complications, NYHA IV functional class 90 days after PVL closure, NYHA improve at 90 days and follow-up and red blood cells transfusion (yes/no) in follow-up.

**Supplementary Table S3: Predictors of death during long-term follow-up:**

| | P value | HR | 95% Conf. Interval |
|---|---|---|---|
| 3 months HF previous admission | 0.000 | 7.0 | 2.7 – 16.5 |
| NYHA IV functional class 90 days after PVL closure | 0.030 | 4.5 | 1.2 – 17.4 |
| Improve NYHA class at follow-up | 0.004 | 0.2 | 0.1 – 0.6 |
| HF vs HA indication | 0.000 | 6.7 | 2.7 – 16.6 |

Univariate analysis: Previous CAD, basal NYHA, 3 months HF previous admission, previous red blood cells transfusion (yes/no), basal hemoglobin, basal LDH, STS score, EuroScore I, HF vs HA indication, PVL location, technical success, procedural success, procedural complications, in-hospital complications, NYHA improve at 90 days and follow-up, NYHA IV functional class 90 days after PVL closure and red blood cells transfusion (yes/no) in follow-up.

**Supplementary Figure S1**

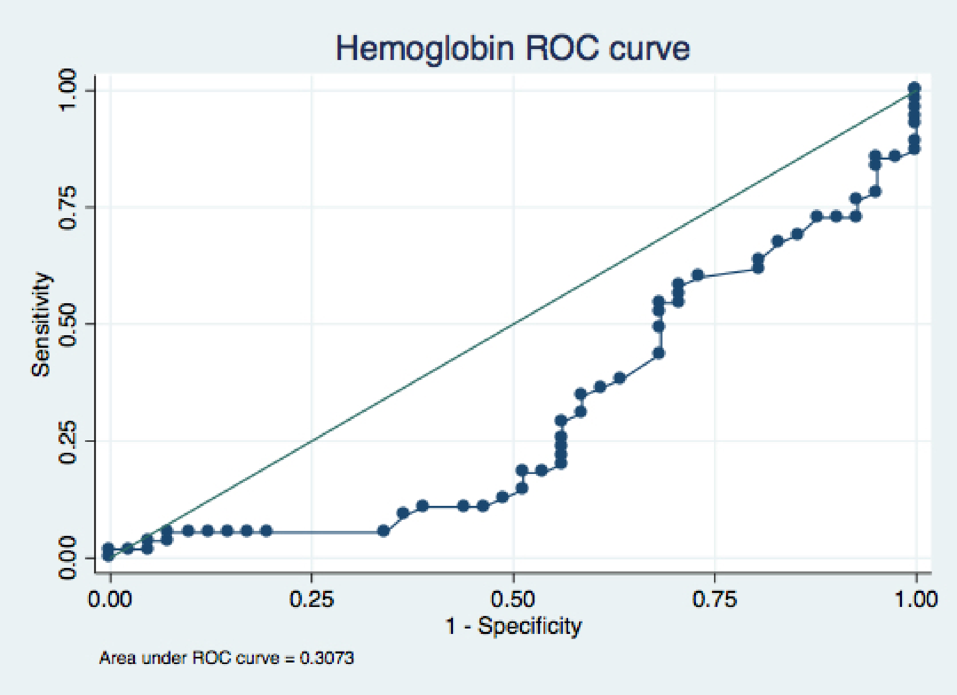

## Supplementary Figure S2

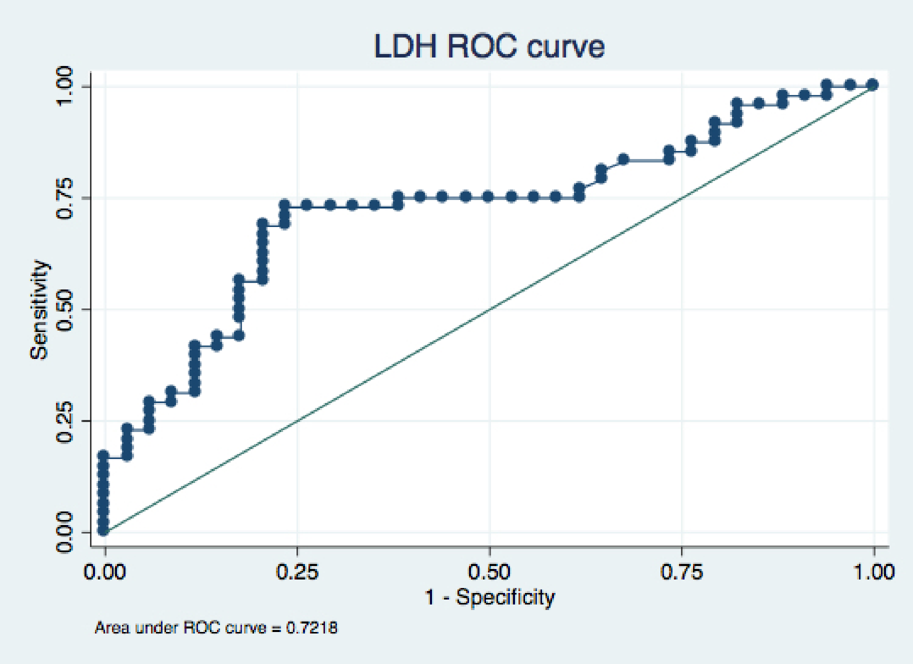

Supplement: Supplementary file 1 [file jcm-11-04835-s001.zip › jcm-1822189-supplementary.pdf]
